# 360-degree Delphi: addressing sociotechnical challenges of healthcare IT

**DOI:** 10.1186/s12911-020-1071-x

**Published:** 2020-06-05

**Authors:** Heiko Waldmüller, Cord Spreckelsen, Hannah Rudat, Norbert Krumm, Roman Rolke, Stephan Michael Jonas

**Affiliations:** 1 Department of Medical Informatics, Uniklinik RWTH Aachen, Aachen, Germany; 2 Faculty of Medicine, University of Wuerzburg, Würzburg, Germany; 3 Institute of Medical Statistics, Computer and Data Sciences, Jena University Hospital, Jena, Germany; 4 Department of Palliative Medicine, Medical Faculty RWTH Aachen University, Aachen, Germany; 5 Department of Informatics, Technical University of Munich, Munich, Germany

**Keywords:** Delphi technique, 360-degree Delphi, Stakeholder, Project planning, Feedback, Medical informatics, Consensus, Quality improvement, Qualitative research, Health information management, Group processes

## Abstract

**Background:**

IT systems in the healthcare field can have a marked sociotechnical impact: they modify communication habits, alter clinical processes and may have serious ethical implications. The introduction of such systems involves very different groups of stakeholders because of the inherent multi-professionalism in medicine and the role of patients and their relatives that are often underrepresented. Each group contributes distinct perspectives and particular needs, which create specific requirements for IT systems and may strongly influence their acceptance and success. In the past, needs analysis, challenges and requirements for medical IT systems have often been addressed using consensus techniques such as the Delphi technique. Facing the heterogeneous spectrum of stakeholders there is a need to develop these techniques further to control the (strong) influence of the composition of the expert panel on the outcome and to deal systematically with potentially incompatible needs of stakeholder groups.

This approach uses the strong advantages a Delphi study has, identifies the disadvantages of traditional Delphi techniques and aims to introduce and evaluate a modified approach called 360-Degree Delphi. Key aspects of 360-Degree Delphi are tested by applying the approach to the needs and requirements analysis of a system for managing patients’ advance directives and living wills.

**Methods:**

360-Degree Delphi (short 360°D), as a modified Delphi process, is specified as a structured workflow with the optional use of stakeholder groups. The approach redefines the composition of the expert panel by setting up groups of different stakeholders. Consensus is created within individual stakeholder groups, but is also communicated between groups, while the iterative structure of the Delphi process remains unchanged. We hypothesize that (1) 360-Degree Delphi yields complementary statements from different stakeholders, which would be lost in classical Delphi; while (2) the variation of statements within individual stakeholder groups is lower than within the total collective. A user study is performed that addresses five stakeholder groups (patients, relatives, medical doctors, nurses and software developers) on the topic of living will communication in an emergency context. Qualitative open questions are used in a Delphi round 0. Answer texts are coded by independent raters who carry out systematic bottom-up qualitative text analysis. Inter-rater reliability is calculated and the resulting codes are used to test the hypotheses. Qualitative results are transferred into quantitative questions and then surveyed in round 1. The study took place in Germany.

**Results:**

About 25% of the invited experts (stakeholders) agreed to take part in the Delphi round 0 (three patients, two relatives, three medical doctors, two qualified nurses and three developers), forming a structured panel of the five stakeholder groups. Two raters created a bottom-up coding, and 238 thematic codes were identified by the qualitative text analysis. The inter-rater reliability showed that 44.95% of the codes were semantically similar and coded for the same parts of the raw textual replies. Based on a consented coding list, a quantitative online-questionnaire was developed and send to different stakeholder groups.

With respect to the hypotheses, Delphi round 0 had the following results: (1) doctors had a completely different focus from all the other stakeholder groups on possible channels of communications with the patient; (2) the dispersion of codes within individual stakeholder groups and within the total collective – visualized by box plots – was approximately 28% higher in the total collective than in the sub-collectives, but without a marked effect size. With respect to the hypotheses, Delphi round 1 had the following results: different stakeholder groups had highly diverging opinions with respect to central questions on IT-development. For example, when asked to rate the importance of access control against high availability of data (likert scale, 1 meaning restrictive data access, 6 easy access to all data), patients (mean 4.862, Stdev +/− 1.866) and caregivers (mean 5.667, Stdev: +/− 0.816) highly favored data availability, while relatives would restrict data access (mean 2.778, stdev +/− 1.093). In comparison, the total group would not be representative of either of these individual stakeholder needs (mean 4.344, stdev +/− 1.870).

**Conclusion:**

360-Degree Delphi is feasible and allows different stakeholder groups within an expert panel to reach agreement individually. Thus, it generates a more detailed consensus which pays more tribute to individual stakeholders needs. This has the potential to improve the time to consensus as well as to produce a more representative and precise needs and requirements analysis. However, the method may create new challenges for the IT development process, which will have to deal with complementary or even contradictory statements from different stakeholder groups.

## Background

Healthcare IT projects have critical sociotechnical implications, including challenging ethical, organizational and legal issues. As a consequence, their success depends on multi-disciplinary efforts in exploring project opportunities, eliciting and prioritizing requirements and agreeing preconditions for IT applications. In the past, several systematic approaches such as Delphi studies, focus groups and nominal group processes, have been used to support the requirements specification, consensus, and evaluation of healthcare IT projects [1]. Special sociotechnical implications with many involved parties can limit these approaches as individual needs might not be represented in a group consensus.

Healthcare IT projects tend to accumulate the same problems that are well-known in IT projects in general: the famous (although controversial) CHAOS report and other IT surveys have revealed that many projects are started but then cancelled (15.52% in 2005, 11.54% in 2007) or perform poorly [2–5]. A major reason behind these failures is the abundance of requirements and scope changes [3], while stakeholder involvement and user inclusion during the project process have proved to be critical for success [6, 7]. This is especially true in clinical systems: a systematic investigation of 40 scientific articles identified the inclusion of all stakeholders in the implementation process as a success factor in 24 out of the 40 articles [8]. Thus, it is necessary to create the possibility of coping with the complexity of multiple stakeholders [9].

The Delphi technique (DT) is a well-established iterative survey technique for gathering information or reaching consensus in a collective [10]. The technique combines qualitative and quantitative data elicitation methods. Developed in 1963 by the American RAND Corporation and named after the oracle of Delphi [11], the DT was originally used for military decision-making. Nowadays it is widely used in medicine and healthcare [1, 6]. The main advantage of this method is that it can find a consensus in a situation of uncertainty or when there is a lack of empirical evidence.

During an investigation into the communication of advance directives and last wills through web-based technologies, several shortcomings of the Delphi technique became apparent. Implementation of emergency and end-of-life patient data is a project of potentially national moment that can easily be challenged by any party using the system. It is therefore essential to include all groups of stakeholders and to cover all user needs appropriately. Advance directives and last wills are used or needed by both laypeople and healthcare professionals. Thus, user needs vary from the simplified creation of documents to the assisted interpretation of a living will. It has been reported that the choice of the expert group can have a strong influence on the DT outcome [12]. In a traditional expert group, aspects could be underrepresented because of the selection of the study collective. Stakeholder groups within the total collective might have incompatible needs for which agreement cannot be found, and this could lead to a watered-down version of a consensus [10].

The problem of creation and distribution of end-of-life health information is indeed a major problem worldwide. Since the study took place in Germany, we want to put the topic into national perspective. Christ et al. showed in a patient study that only 27.8% out of 496 people in an emergency department in Germany with an average age of 65 years (two surveys at different time points) had an advance directive or living will. Only 3.2% could present their files to the health care professionals. The authors concluded a higher acceptance in the elderly population, although hardly any consequences were observed in daily operations because of the lack of documents [13]. A study conducted in 2017 by Kluge et al. with longer timeframe has shown that 51.3% of 998 patients had at least one document (either advance directive or patient will). Out of the 998 patients 39.6% stated to have handed the documents to the health care professionals. Only 23% of these were integrated in the digital health records of the hospital. In conclusion, out of 998 patients 8.5% empowered the health care professionals to decide in their interest in case of an emergency [14]. The topic of end-of-life health information is a topic needed to be solved, because the current situation is insufficient, for example documents already created do not reach the decision maker. Further investigation shows that it is a worldwide issue having not enough end-of-life-decision documents with national differences [15]. Since 01.09.2009 the German “Patientenverfügungsgesetz”, the advance decision law, is in operation. A detailed situation analysis was performed in in 2011 [16, 17]. It indicated that lack of time plays an important role in emergency situations. An emergency doctor might not have the time required to talk to the patient’s representative, to search for documents or to call relatives.

Thus, the aim of this manuscript is to introduce a modification of the traditional Delphi technique to address the issues described above. Instead of one group of pre-selected experts, a structured expert board of system-stakeholders is surveyed. This means that practical and diverse statements will be made and can be incorporated into the project. Additionally, while a single collective is investigated, stakeholder groups with different interests in the project are created and evaluated separately. Specifically, (a) patients, (b) relatives, (c) doctors, (d) nurses and (e) developers are selected as different stakeholders. Using this approach, we are able to include opinions and statements that would not be covered using a single expert collective as is traditionally done. Consequently, the following hypotheses are formulated:

- **Hypothesis 1:** Using the new DT design, complementary statements from different stakeholder groups can be collected that would be lost if experts were chosen from only one or a few stakeholder groups.
- **Hypothesis 2:** The opinion of individual stakeholder groups has a lower dispersion than the opinion of the total collective; that is, the variation of statements within individual stakeholder groups is lower than it is within the total collective.

Both hypotheses will be evaluated on the basis of the users’ unbiased statements and opinions. Hypothesis 2 could be proven on a quantitative round. However, a stronger point can be made if the dispersion can be shown within answers to open questions rather than within the fixed scale items of a quantitative questionnaire. Thus, the evaluation is performed on the results of the qualitative Delphi round, and the study participants are not influenced during the consensus phases or required to limit their answers to fixed scales.

## Method

**Fig. 1:**
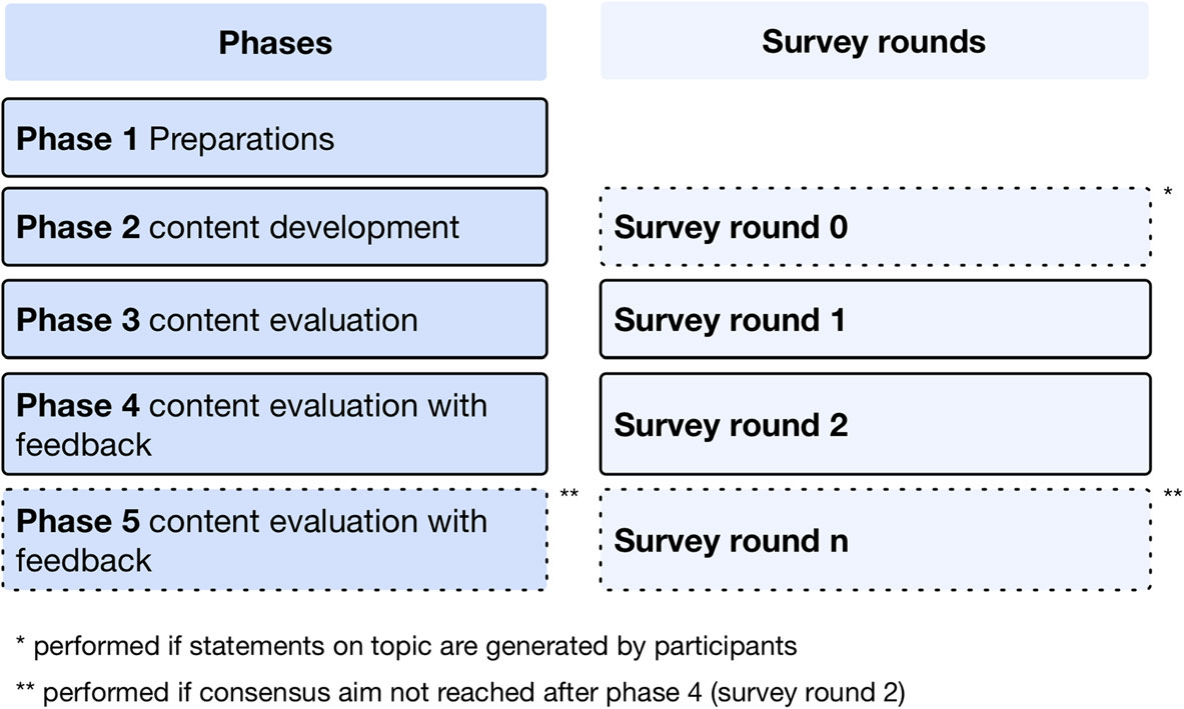
Phases and corresponding survey rounds

The traditional Delphi method consists of five phases: preparation (Phase 1), content development on the relevant topic with an optional so-called round 0 survey (Phase 2), initial content evaluation based on statements in a round 1 survey (Phase 3), re-evaluation of content statements with feedback of all participants in a round 2 survey (Phase 4), and, if necessary, repeats of the pattern of re-evaluation until a consensus is reached (Phase 5) (Fig. 1). The participants in the survey rounds are experts selected by the science or monitoring team. The definition of an expert in a certain area is subjective and finding the consensus can be influenced by the selection of the experts or the number of iterative rounds in the study [14].

### 360-degree Delphi design – what’s new?

**Fig. 2:**
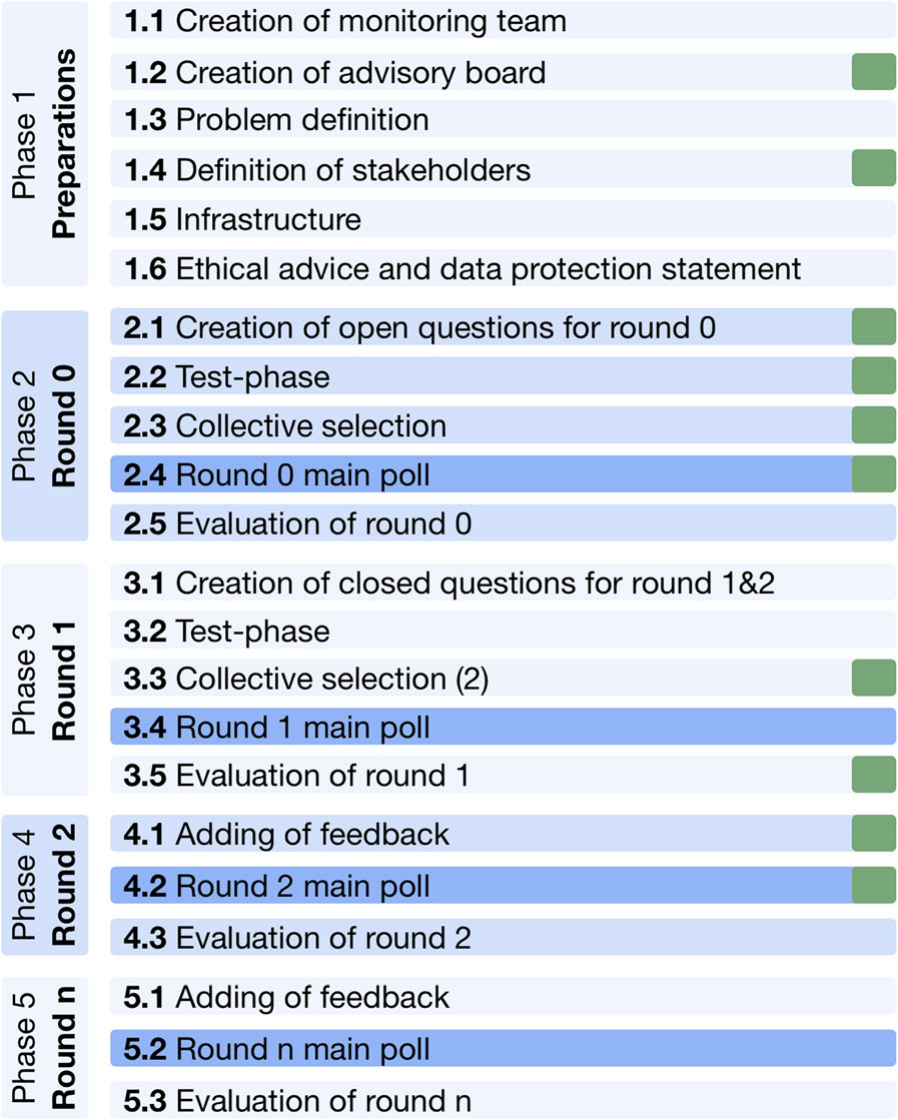
Structure of the 360-Degree Delphi (360°D) approach. Changes to the traditional Delphi technique and items that are now mandatory are marked with a green mark

The new approach is called 360-Degree Delphi (360°D for short) to represent the aspect that a collection of opinions is gathered. The name is a reference to 360-Degree feedback, in which the competence or performance of personnel is evaluated not only by their supervisors or managers, but also by their co-workers and subordinates. In a similar way, 360°D evaluates aspects of a topic by considering the opinion of all relevant stakeholders. Another main innovation of the 360°D approach, compared to the conventional DT, is the composition of the expert collective, which follows a practically-oriented stakeholder principle. To differentiate the stakeholder groups, the criteria for inclusion need to be precisely defined. Experts are usually selected according to the number of their publications, their prior work in the field under investigation or the interest they have shown in the specific topic, although there are other methods. With the 360-Degree approach, people are selected on the basis of their participation in the domain. In other words, they are experts as the result of their daily roles and work in the area that will be influenced by the findings of the study. Another innovation is the mandatory performance of certain DT components that are usually voluntary (Fig. 2).

In a similar way to traditional DT, 360°D is structured in five main phases, but some aspects are partially modified (Fig. 2): (Phase 1) preparation phase, (Phase 2) qualitative round/questionnaire, (Phase 3) first qualitative round, (Phase 4) first consensus round, and (Phase 5) optional repetitions of Phase 4. Only changes in the sub-tasks performed are reported in the following sections: we refer readers to the online supplements for a complete itemized task description.

#### Phase 1 – preparation phase

The preparation phase consists of six steps, (1.1) the creation of the monitoring team, (1.2) the creation of the advisory board, (1.3) the definition of the scientific problem, (1.4) the definition of the stakeholder groups, (1.5) the creation of the survey infrastructure, and (1.6) the obtaining of ethical approval. Steps 1.1, 1.3 and 1.5 are performed in exactly the same way as in the traditional DT.

##### Step 1.2. Creation of the advisory board

In 360°D the founding of an advisory board is mandatory. The advisory board does not conduct the study, but it gives scientific assistance to minimize bias by the monitoring team. It is especially important in the 360°D approach to review the definition of the stakeholder groups [15]. The people involved in this board are science-related, not necessarily topic-related, and are experienced in evaluating scientific projects. The advisory board accompanies the study by meeting the monitoring team over the course of the study. Since the advisory board is not directly involved in the work, advantages and disadvantages can be identified and notified to the monitoring team.

##### Step 1.4. Definition of stakeholder groups

The principal new step in 360°D is the definition of stakeholder groups; the purpose here is to categorize the participants on the basis of their different (possibly corporate) interests in or views on the defined problem. In 360°D the term “participant” is used for any member of the study collective (which is the expert panel in classical DT). In finding a consensus regarding a certain topic, the participants’ current fields of interest and work should be related to that topic.

##### Step 1.6. Ethical advice and data protection statement

Ethical approval from an institutional review board or ethics committee and a statement or certificate by a privacy officer are recommended for the 360°D approach as part of good scientific practice. Ethical approval will be mandatory if the data acquired are related to individuals, or if there are questions related to sensitive topics such as infectious state, social status or financial status. Similarly, data protection is critical not only for ensuring data quality but also to increase compliance by the study participants.

#### Phase 2 – qualitative round

Phase 2 (or round 0) links five sub-steps: (2.1) creation of open questions, (2.2) test-phase, (2.3) collective selection, (2.4) main poll, and (2.5) evaluation.

##### Step 2.1 creation of open questions

Since the goal of the first question-phase is the collection of general knowledge and problem statements from a stakeholder perspective, open questions for qualitative data acquisition are formulated by the monitoring team using standard question design methods (e.g. [16, 17]).

Using the defined problem and the defined stakeholder groups, the wording of the questions is adjusted to make them understandable by the stakeholder groups. Therefore, questions addressed to particular stakeholder groups might be formulated differently. The level of abstraction and the content should be the same for every stakeholder group. A change of perspective can help stakeholders to provide answers based on their role. For example, a question from a patient’s perspective could be “How would you ***provide*** your will in an emergency situation?”, while the analog from the perspective of a medical doctor could be “How would you ***gather*** the patient’s will in an emergency situation?”

##### Step 2.2 test-phase

Before the main poll, the qualitative open questions are tested to see whether they are understood correctly or whether editing is necessary. Participants in this test-phase should not be included in the later rounds. The questions can be tested either in face-to-face interviews or by a pre-survey created for test purposes. A major advantage with oral interviews is that direct and indirect feedback can be obtained from the participants.

##### Step 2.3. Collective selection

In addition to the creation of the survey, the monitoring team selects the participants. Participants are selected on the basis of a practically oriented stakeholder principle: their practical experience in the field of study, their possible future interaction with the system, or their current usage of similar systems. Each stakeholder group should contain approximately the same number of participants. It is recommended that a bigger pool is contacted, as the response rate is approximately 25% and the number of participants should not be less than ten for a qualitative collective performing a qualitative study [18].

##### Step 2.4 Main poll

The main quantitative poll is performed in the same way as round 0 with the general DT. However, depending on the questions, appropriately formulated questionnaires might be necessary for each stakeholder group (compare Step 2.1).

##### Step 2.5 evaluation

The answers to the open questions consist of qualitative text data. To evaluate the results, a systematic approach is used. Some variants for qualitative data evaluation are available from social studies. Well-known techniques are “Grounded Theory” [19] or “Qualitative Content Analysis” [20, 21]. To reduce the subjectivity of the qualitative analysis, multiple analyses by different people from the monitoring team can be performed independently and merged, providing the opportunity to assess inter-rater reliability. Performing the analysis separately for each stakeholder group is important, even if similar or almost identical questionnaires are used for all stakeholder groups. A statement catalog is created using, for example, a coding system based on the statements for each stakeholder group and in total. Grouped by codes derived by bottom-up coding (see [21]) and agreed by the raters, the catalog of statements provides the basis for creating quantitative items for the following rounds.

#### Phase 3 – first quantitative round

Phase 3 is the starting point for deriving consensus in phases 4 and 5. The participants score the items derived from the qualitative phase. The sub-steps of this round are similar to a traditional DT: (3.1) creation of closed questions for rounds 1 and 2, (3.2) test-phase, (3.3) collective selection (2), (3.4) round 1 main poll and (3.5) evaluation of round 1. Steps 3.2, 3.3, and 3.4 are performed as in traditional DT, with the collective being selected using the same practically oriented stakeholder principle as in Step 2.3. A new collective can be formed or persons can be added to the existing collective.

##### Step 3.1. Creation of closed questions for rounds 1 and 2

In this step the statement catalog from Step 2.5 is used to generate quantitative questionnaires by, for example, systematically transforming statements into Likert-scale items or priority rankings. Statements are selected according to their importance within the total collective but also within individual stakeholder groups. This guarantees that statements that are important to only one stakeholder group will still be reflected in the total consensus, even if this group is small or would have a low impact otherwise. The remaining process is performed as in traditional DT.

##### Step 3.5 evaluation

The data collected in round 1 is quantitative data, so it can be analyzed by descriptive statistics, as in ordinary DT studies or any other survey. The choice of the analytical approach depends on the level of measurement chosen. A new aspect of 360°D is that data are aggregated for each stakeholder group separately instead of overall figures being generated as in traditional Delphi studies. The results are documented accordingly.

#### Phase 4 – first consensus round

In Phase 4 a consensus may appear. The following steps are conducted to perform phase 4: (4.1) adding of feedback, (4.2) round 2 main poll and (4.3) evaluation of round 2. Steps 4.2 and 4.3 are performed in an identical way to Steps 3.4 and 3.5. If possible, the collective is not altered.

##### Step 4.1 addition of feedback

The evaluation results of each statement for each stakeholder group are communicated in an understandable way to the total collective. For example, the average evaluation and dispersion of each stakeholder group for each question is displayed [18, 22, 23]. Feedback influences the responses of the participants towards a consensus. Displaying the means and dispersion of ratings for each stakeholder group instead of an overall mean and dispersion allows a faster consensus within stakeholder groups. This may be a benefit of 360°D over traditional DT, as the latter would not reach overall consensus if two stakeholder groups cannot find a common consensus on a statement.

#### Phase 5 – 2nd to *n*th consensus round

All further phases are performed in an identical way to Phase 4.

### Evaluation techniques

The evaluation of Phase 1 of 360°D is performed by looking at several aspects: (1) inter-rater reliability is calculated in order to create a solid coding base, (2) Hypothesis 1 is evaluated using excerpts of the coding catalog, and (3) Hypothesis 2 is evaluated by calculating the dispersion of statements within stakeholder groups. Similarly, statistics from Phase 3 are reported alongside to support the initial findings for Hypotheses 2.

#### Inter-rater reliability

Two coders receive the raw collected data and independently create a coding structure and base. Data are read and the coding base and corresponding statement coding are created in a bottom-up procedure resulting in codes associated with parts of the raw statements. The coding versions by the two coders are compared to check if the bottom-up procedures for each coder show similarities. Code matches between the codes are created based on position in the statement texts and the semantic meaning of the code label. If multiple codings occur at a text position for one coder, but not for the other one, only one code match is created and other codings are labeled as unmatched. Since the code structure and code base are created independently, the semantic meaning of individual codes could vary. Each matched code was therefore presented to three evaluators who rate the code matching either as a semantic match or as a mismatch. The rate of semantically matched codes based on a majority vote scheme (at least two out of three) and single vote scheme (one out of three) is reported in the results.

#### Evaluation of hypothesis 1

To investigate Hypothesis 1, we assume that any of the five stakeholder groups would be eligible to stand as an expert collective for the traditional Delphi technique by themselves. Thus, we analyze whether any of the given statements (represented by their codings) are made exclusively or predominantly by one of the stakeholder groups, thereby being complementary to the other groups.

#### Evaluation of hypothesis 2

The opinion dispersion of the stakeholders is measured using the code repository derived by the raters. Dispersion *D* is defined as the average standard deviation across the different coding occurrences.

$$ D=\frac{\sum_C{\sigma}_c}{N_C} $$

with *C* being the different codes, *N*_*C*_ the number of codes and *σ*_*c*_ the population standard deviation of occurrences within the code.

$$ {\sigma}_c=\sqrt{\frac{\sum_S{\left({x}_{C,s}-\overline{x_C}\right)}^2}{N_S}} $$

with *S* being the different stakeholders within the group, *x*_*C*, *s*_ the number of times the code *C* is associated with stakeholder *S*, $\overline{x_C}$ the average number of times code C is associated with stakeholders in the group, and *N*_*S*_ the number of stakeholders.

Dispersion is reported per stakeholder group and for the total collective using box plots.

In addition, mean and standard deviation are reported and compared for the Phase 3 quantitative questionnaire.

### Software tools

The web-based tool LimeSurvey (Version 2.05, LimeSurvey GmbH, Hamburg, Germany) was used for online questionnaires. The coding and further analysis was supported by MaxQDA (Version 10, VERBI GmbH, Berlin, Germany), Microsoft Excel (Version 2011, Microsoft Corporation, Redmond, WA, USA) and Matlab (Version R2014b, The Mathworks, Natick, MA, USA).

## Results

### Study implementation

In order to verify the hypotheses, a 360°D process was performed at the University Hospital of RWTH Aachen University in the context of advance directives and living will communication. Here, 360°D Phases 1 through 3 are reported to indicate differences of opinion in stakeholder groups.

#### Phase 1 – qualitative phase

##### Step 1.1. Creation of the monitoring team

The monitoring team (HW, SMJ) was assembled and tasks were distributed.

##### Step 1.2. Creation of advisory board

An advisory board (CS, RR) was assembled by the monitoring team. During the first two phases of the study, monthly meetings were held to control the study and verify decisions by the monitoring team.

##### Step 1.3. Problem definition

Little is known about the actual functionality and problems arising in the creation, distribution and interpretation of advance directives. Also, options for the improvement of communications about advance directives between people affected by an emergency situation and helpers are rare. The scientific problem was therefore defined as: “What are the current perceptions AND proposals about exchange of information in non-responsive situations?”

##### Step 1.4. Definition of stakeholders

Criteria for the stakeholder groups were defined. The monitoring team identified five stakeholder groups:

1. **Medical doctors**, who are assumed to act in the patient’s interest and have a valid medical licence by state law. The level of training (resident, consulting physician) was not defined.
2. **Qualified nurses or caregivers**, who have a close relationship with patients and might are the first responder in cases of emergency; A person who is a qualified nurse by law or in training of his / her qualification (2nd year or higher)
3. **Patients**, who need to formulate their will appropriately and select how it should be transmitted if they are unable to do so themselves; A person who has or had an illness and is or was in medical treatment. The severity of illness or affected body system was not defined, however, only patient groups identified through self-help groups of life-threatening diseases such as myocadiac infarction or stroke were approached.
4. **Relatives**, who eventually have to make medical decisions in the absence of an advance directive or lack of direct patient-doctor communication; A relative is in a familiar connection with a patient that matches the criteria of item 3; a relative doesn’t have to have an advance directive on behalf of the patient.
5. **Developers**, who develop and maintain the system. A person who has been trained or is in training of information technology. The level of training was not defined.

Intersections between “patient” and “relative” or any of the others can occur in specific cases, such as a relative of a person with a severe health condition who is also undergoing medical treatment, or a patient who is also a medical professional. Therefore, each participant was instructed to answer as a member of the **most applicable** stakeholder group.

##### Step 1.5. Infrastructure

The contact channel was email, social networks, phone or personal, with a focus on email. The survey infrastructure was primarily online, to allow remote participation and limit interviewer bias.

##### Step 1.6. Ethical advice

Data security was defined by the data protection officer and confirmed: to allow anonymity the monitoring team agreed not to save the IP-addresses, timestamps, or any personal data of the participants. Ethical approval was obtained from the ethics committee of the Uniklinik RWTH Aachen (EK 363–15).

#### Phase 2 – qualitative round

To investigate the difference between the current DT and the novel 360°D, a qualitative survey was performed. Round 0 statements were not set by the monitoring team, but were collected using a qualitative questionnaire to reduce bias.

##### Step 2.1. Creation of open questions

The monitoring team created open questions on different aspects related to the topic (see Addenda A and B). A scenario-based question form was developed to compensate for informational gaps. Additionally, technical terms were either avoided or were defined in the survey. An online survey was created for three different categories: (i) *medical professionals* for doctors and nurses, (ii) *care recipients* for patients and relatives and (iii) *developers* for developers. The main difference between the questionnaires was formulation and introduction to the topic.

##### Step 2.2. Test-phase

The questions were tested using semi-structured interviews. The test respondents were three medically qualified individuals (one doctor and two nurses) who were asked to select and then modify the questions for a better outcome.

##### Step 2.3. Collective definition

In order to find contacts appropriate to the collective, a list of medical institutions was created using the practically oriented stakeholder principle. To create a heterogeneous collective, there was an equal number of institutions with each of the following characteristics: level of care (maximum care institution, specialist care institution, primary healthcare, doctors office, retirement home), institution sponsor (church-related, public, private), speed of emergency reaction (fast, slow), medical area (oncological, internist, geriatric, emergency services), geographical area (rural, urban). Stakeholders were collected associated with the primary contact person (mostly doctors) such as patients of the doctor, the relatives of the primary contact persons patient. Software developers where identified by projects associated with the field of medical informatics. There was no personal relationship between monitoring team and collective.

##### Step 2.4 round 0 main poll

The institutions were contacted by phone or email. A follow-up email, including information on the topic and a printable flyer with access to the online surveys (Short-URL, URL and QR-Code), was sent. Information of the project consisted out of the reason why the study is conducted, to establish an emergency data set for will communication, and a hypothetical use case. The contact person in the identified institutions was asked to print out and hand the flyer to: one patient, one doctor, one nurse and one relative of a patient. The survey was closed in January 2016 with the following numbers of answers: 30 in total, of which ten were complete. There were three in each of the medical doctor and patient stakeholder groups, and two in each of the qualified nurse and relative stakeholder groups. The response rate of the first contact by phone was 80% (*n* = 32), the response rate for completed answers was 25% (*n* = 10). The stakeholder label was selected by the participant during the survey and saved in the survey database. The average time for the participants to complete the survey was 25.87 min. In addition, three developers with experience in medical informatics but not related to the project were surveyed.

##### Step 2.5 evaluation of round 0

The database was converted into a single document for each participant so that data analysis could be performed (HW). The documents were named with a database ID and grouped according to the indicated type of stakeholder. Qualitative coding was performed for the full collective and the stakeholder groups with an inductive category development (bottom-up coding) [24]. Two coders performed the analysis independently using two different systems and compared their results (HW, NK). Both the coding and the coding structure were created independently. Rules for creating the categories were defined to evaluate similar content structures. The coding bases were subsequently consented and brought into a joined format.

##### Inter-rater reliability

Codings were matched and inter-rater reliability was calculated. In total, 238 codes were identified, of which 158 could be matched by their location. Out of the 158 matched codes, 107 (44.95% of total codes, 67.72% of matched codes) were evaluated as semantically similar in a majority-vote scheme, and 132 (55.46 and 83.54% respectively) in a single-vote scheme by three evaluators. In other words, 44.95% of the codes were created at the same position in the raw text, and two out of the three independent evaluators rated the codes given to these positions as semantically similar. Major sources of differences were variability in coded text passages, consistent coding of suggestions that occurred on multiple occasions, and level of abstraction. The coding and coding structure were then unified and agreed upon by the two coders to form a coding base for future use.

#### Phase 3 – first quantitative round (traditional Delphi: round 1)

##### Step 3.1 creation of closed questions for round 1

Based on the coding base, questions were identified and transformed into a quantitative survey (HR) (see Addendum C). The questions were divided into A “simple questions” and B “matrix questions”. Matrix questions are used to weigh different, potentially conflicting aspects against each other. In this case, privacy and data security, and ease of accessibility and change of data was weighted against each other, as they are often mutually exclusive in a system. For example, if personal data is stored encrypted using a password it is less prone to breach of privacy, but also not easily accessible in case of emergency. Especially in these topics the sub-groups did not match in the Phase 1 and 2 questionnaires.

##### Step 3.2 test-phase

The questions were tested by scientists associated and non-associated to the 360 degree project. Markers of the test were (1) understanding questions, (2) duration filling out the survey, (3) selection among ordinal and other scales (SMJ/HR/HW).

##### Step 3.3 collective selection (2)

Sub-groups were contacted on different channels: patients and relative associations via social platforms and support groups, doctors and caregivers via hospitals, IT-developers over direct contact and phone calls. (HR, HW). To access the survey a link for access over the internet was used.

##### Step 3.4 round 1 main poll

The questions were converted into a single document for each participant so that data analysis could be performed (HR). The online survey was opened from Febuary 1st 2018 till October 10th in 2018. In total 62 persons participated with 60 persons (11 doctors, 6 caregivers, 9 relatives, 28 patients and 6 IT-developers) completing the questionnaire. The 2 uncompleted persons stopped filling out the form because of connection loss to the data server.

### Hypothesis 1

Several instances were found in which statements were made exclusively or predominantly by just one of the stakeholder groups. The analysis regarding the channels for will communication in an emergency situation (Questions 1 through 4, Addenda A/B) show that the stakeholder groups of patients, relatives and qualified nurses named speech and gesture (i.e. direct contact) as possible options, in 12 statements. In contrast, doctors only once named direct contact with the patient as a possible channel (Table 1).

**Table 1:** Numbers of options for direct will communication in emergency situation per stakeholder group

Stakeholder Group	Code		
	*Speech*	*Gesture*	*Direct Contact*
Patients	Count: 3 “*… if responsive by conversation.”*	Count: 1 “*… by gesticulation.”*	Count: 0
Relatives	Count: 2 *“speaking”*	Count: 3 “*… by alternative communication.”*	Count: 0
Doctors	Count: 0	Count: 0	Count: 1 *“check, if possible to contact patient.”*
Caregivers	Count: 2 *“Verbally.”*	Count: 1 “*… nonverbal communication …*”	Count: 0
Developers	Count: 0	Count: 0	Count: 0

Indirect contact shows similar characteristics: doctors value relatives as a source of information, and were the only stakeholder group with seven out of a total of eight statements with this coding (Table 2).

**Table 2:** Options for indirect will communication in emergency situation

	Patients	Relatives	Doctors	Caregivers	Developers
Code	Amount	Amount	Amount	Amount	Amount
**Text**	2	0	0	0	1
**Patient’s will**	3	1	5	3	2
**Organ donor card**	0	2	0	0	2
**Advance directive**	1	0	2	1	1
**Presumption**	0	0	3	0	0
**Relative**	0	0	7	1	0
**Other**	0	0	2	0	0

General knowledge of advanced directives exists in the doctor, patient, developers and caregiver stakeholder groups, whereas relatives do not mention advance directives. In multiple responses by relatives, the term organ donation card appeared as a named form of will communication, whereas this term was not present in the other groups. Developers did not mention speech, gesture or direct contact at all. In terms of indirect will communication text, patient’s will, organ donor card and advance directives were mentioned.

Any collective made of just one stakeholder group would therefore have missed at least one aspect brought up by another stakeholder group. This proves Hypothesis 1.

### Hypothesis 2

**Fig. 3:**
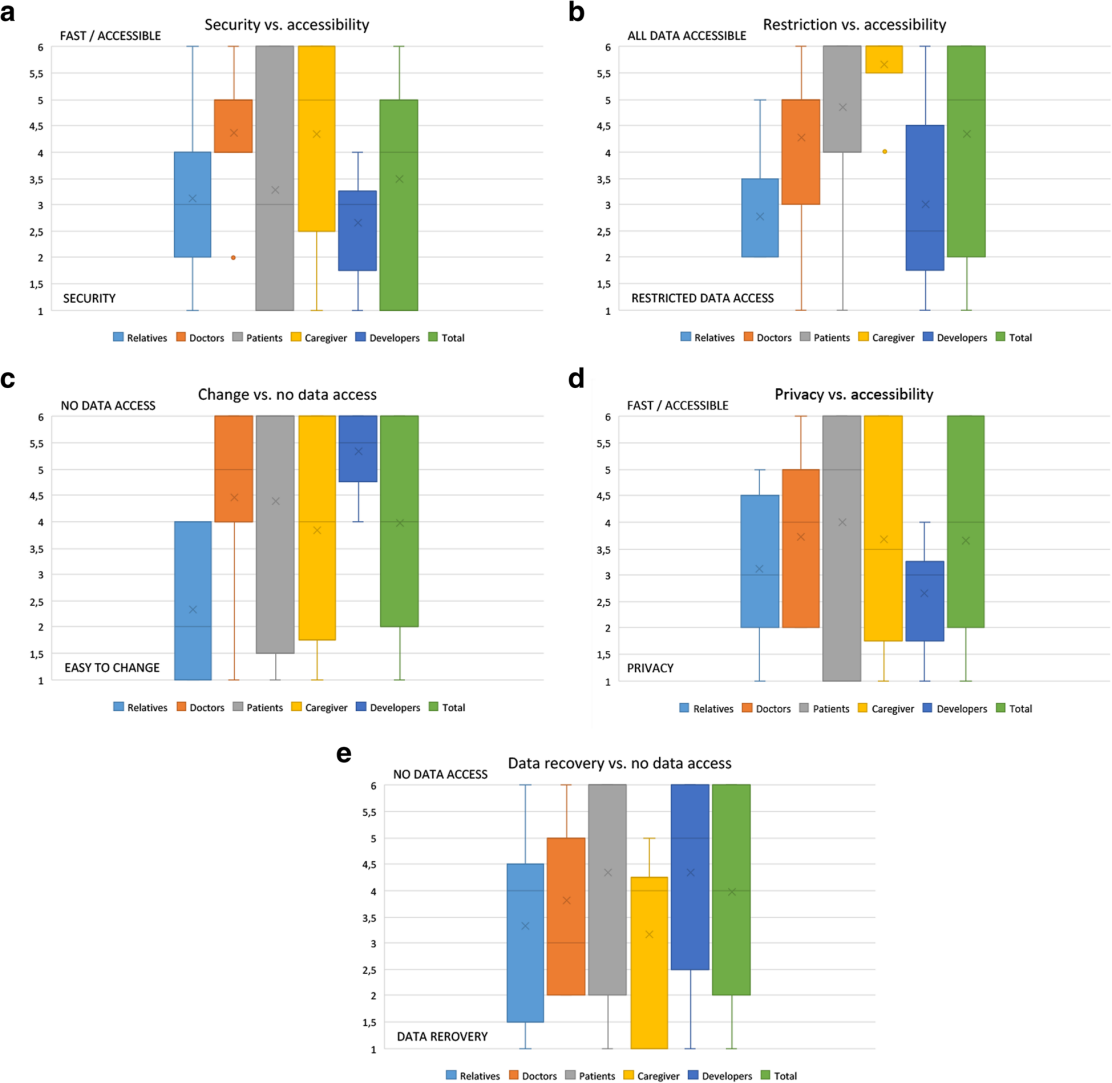
**a** Security vs accessibility, likert-scale answers of the stakeholder groups. **b** Restriction vs. accessibilty, likert-scale answers of the stakeholder groups. **c** Change vs. no data access, likert-scale answers of the stakeholder groups. **d** Privacy vs. accessibilty, likert-scale answers of the stakeholder groups. **e** Data recovery vs. no data access, likert-scale answers of the stakeholder groups

Four Phase 1 and 2, the standard deviation across the population of the occurrences of codings is calculated as a measure of dispersion. It is calculated for each code and for each stakeholder group and the total collective (Fig. 3). Two questions were removed because they were unspecific (Question 7 “Do you have any other aspects to mention?” and Question 8 “What is your opinion of the project?”). The average standard deviation as a measure of dispersion is highest in the total collective. On average, the dispersion is 27.8% higher in the total collective than in the individual stakeholder groups but without a marked effect size.

**Fig. 4:**
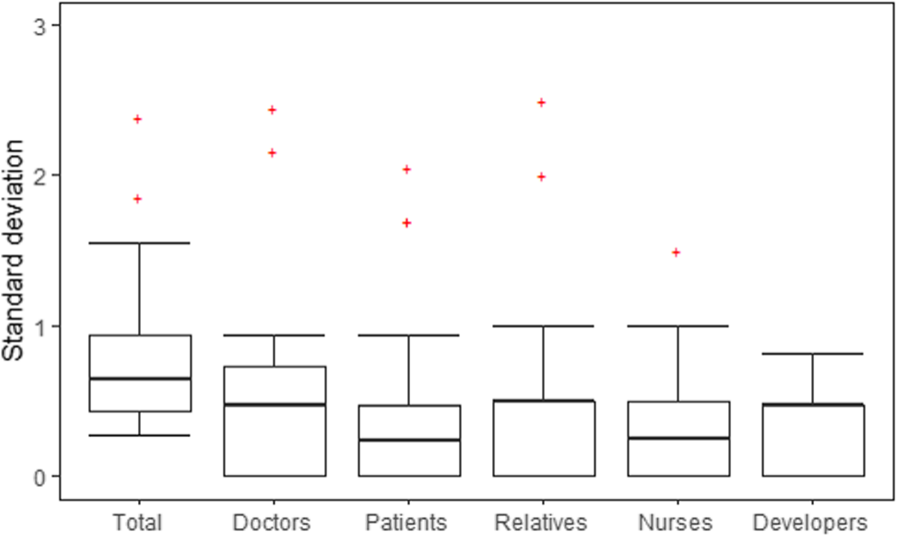
Comparison of dispersion within total collective and individual stakeholder groups in Round 0

Similarly, statistical analysis was performed for each question of the Round 1/Phase 3 questionnaire (SMJ, HW). Results are reported as Box-plots (Fig. 4). Due to the small sample size and study setup, no inferential statistics (T-Test, etc.) were performed but descriptive statistics are reported. While most sub-groups overlapped in their replies, several questions were answered almost mutually exclusive between sub-groups (Fig. 4, Table 3). For example, caregivers and patients were in strong favor of easy access to all data in case of emergency (mean 5.667, Stdev: +/− 0.816 and mean 4.862, Stdev +/− 1.866 respectively), Likert scale, 1 means restrictive data access, 6 easy access to all data), while relatives preferred a more restrictive data access (mean 2.778, stdev +/− 1.093). Since the standard deviation is low in the sub-groups, this question could already be accepted as consented. In comparison, the total distribution across all groups is not consented (mean 4.344, stdev +/− 1.870). Similar effects can be seen in most matrix questions (see Table 3), which supports Hypothesis 2.

**Table 3:** Metrics for questions

	Metric (mean and standard deviation)					
Question	Relatives	Doctors	Patients	Caregiver	Developers	Total
Q1 (Fig. 4a)	Mean: 3.1 SD: 1.453	Mean: 4.4 SD: 1.362	Mean: 3.3 SD: 2.234	Mean: 4.3 SD: 2.066	Mean: 2.7 SD: 1.033	Mean: 3.5 SD: 1.920
Q2 (Fig. 4b)	Mean: 2.8 SD: 1.093	Mean: 4.3 SD: 1.793	Mean: 4.9 SD: 1.866	Mean: 5.7 SD: 0.817	Mean: 3 SD: 1.789	Mean: 4.3 SD: 1.870
Q3 (Fig. 4c)	Mean: 2.4 SD: 1.414	Mean: 4.5 SD: 1.635	Mean: 4.4 SD: 2.227	Mean: 4.4 SD: 2.137	Mean: 5.3 SD: 0.816	Mean: 4.0 SD: 1.953
Q4 (Fig. 4d)	Mean: 3.1 SD: 1.453	Mean: 3.7 SD: 1.421	Mean: 4 SD: 2.187	Mean: 3.7 SD: 2.066	Mean: 2.7 SD: 1.033	Mean: 3.7 SD: 1.870
Q5 (Fig. 4e)	Mean: 3.3 SD: 1.732	Mean: 3.8 SD: 1.601	Mean: 4.3 SD: 2.176	Mean: 3.2 SD: 1.722	Mean: 4.3 SD: 1.966	Mean: 4.0 SD: 1.954

### Additional system design considerations

From all the participants (*n* = 59) in the second round 47.46% (*n* = 28) were patients, 16.95% (*n* = 10) doctors, 15.25% (*n* = 9) relative, 10.17% (*n* = 6) caretakers and 10.17% (*n* = 6) software developers. 26 had created an advance directive. 83% knew about the possibility of an advance directive and donation card, 70% about health care proxy. 44% have an advance directive of their own. Willingness to upload documents online is surprisingly with an average of 94.92% advance directive, 80% emergency card, 78% organ donations, 61.02% vaccination pass and 47.5% personal message. Lowest acceptance has testimonial with 37%.

For practical reasons for how to design a system the collective was questioned. In terms of how to create documents we asked if the participants prefer questionnaire or free text. A majority with 86.44% agreed on questionnaire.

62.71% agreed on a likert scale with 4 items to “agree fully” with “I wish, there would be an easier way than right now to communicate the patient’s interest.

98.31% would create a data set, mostly for themselves (83.05%), but also for their parents (57.63%). Kids and other relatives ranked lowest.

Participants rated, that the data set should be accessible by doctors (100%), the patient himself (76%), relatives (70%) and caretakers (46%). “Everybody” was selected by only 2% of participants. This concludes, that an authorization system is key to a success.

Data changes and management were seen by 89% of patients and 56% of doctors primarily the patients responsibility. 62.71% selected to update the date in 6 months, every year or less often. Time amount per month for content care is mostly selected “a few minutes” (74.48%).

## Discussion

An implementation of the first three phases of the 360°D approach for the elicitation of the requirements for a medical IT project (a system for the communication of advance directives or last wills) was performed. The total collective was composed of five stakeholder groups, each having its own requirements and expectations. The first and second questionnaire rounds (traditionally known as Round 0 and Round 1) were completed and the evaluations were made. Two people, working independently, encoded the qualitative answers, creating an independent coding structure. Given the vast potential variety of the categories (codes) that were developed freely and independently by the raters, a high inter-rater reliability was achieved with a matching rate of semantically similar codes of 44.95% based on a majority vote from three independent evaluators.

The investigation yielded observations supporting the two hypotheses regarding the complementarity and focus of the different stakeholder groups. In the first case, it could be observed that none of the individual stakeholder groups was able to mention all the aspects named by the total collective of all stakeholder groups. In particular, traditional expert collectives such as medical doctors had a unique viewpoint with regards to the direct and indirect communication of the patient’s will.

The approach did not allow inferential statistics to be applied, since this would have required a randomized quantitative study design. Thus, statistical significance cannot be attributed to the results. Nonetheless, the descriptive statistics revealed a trend concerning the dispersion of the statements, which turned out to be lower within each individual stakeholder group than in the total collective. In combination, the two hypotheses might even indicate larger problems with the traditional DT. Statements made by small or underrepresented stakeholder groups might not get attention in expert panels, as the focus might be dominated by the strongest stakeholder group in the panel. These results might be an explanation for the influence that the selection of the experts plays in Delphi studies [14]. The main limitation of this study is the small number of participants. However, a recent study in Nature Human Behaviour by Navajas et al. showed, that combining as few as four consensus choices outperformed the wisdom of thousands of individuals [25]. Only very few patients and relatives could be found, despite the high number of invitations to participate in Phase 2. One explanation might be the intimacy of the topic of advance directive, as it is closely linked to the topic of death and loss. The study was conducted in German, hence we suggest validating the results in other languages and health care cultures in the future. Another limitation of the 360°D method is the inherent consensus per stakeholder group. One has to redefine the concept of consensus for the analysis of the user needs, as no single consensus will be found that can guide development priorities: one consensus for each stakeholder group has to be considered. This shifts the burden of finding a final consensus to the study team, but gives them more information and might lead to solutions that are better tailored towards the target audience and not only towards the expert panel. Partitioning the expert board into stakeholder groups decreases the heterogeneity of each group, which needs careful consideration: past findings regarding the assembling of the expert collective show that heterogeneous collectives are more likely to find highly acceptable solutions than homogeneous ones [26, 27]. 360°D can be assumed to compensate for this effect by informing the (relatively homogeneous) stakeholder groups about the consensus reached by other groups during the Delphi process. Nonetheless, this aspect needs further investigation. Also, since the process of data base technology, world wide web and fast computing we think, that there are no limitations considering the processing of big data amounts as it used to be in the past. Printed lists and paper war are history and new possibilities for data evaluation should be applied to techniques like the Delphi method.

The awareness regarding data protection is relatively high in Germany compared to other countries. It has been shown, that the average amount of money willing to spend for data protection is ranked higher in Germany than in other countries [28, 29]. Therefore, data protection was particularly surveyed to the collective.

## Conclusion

Our results support the assumption that the new 360°D approach has the potential to overcome certain drawbacks of the traditional DT by defining the expert collective more clearly and aiming for more heterogeneous stakeholder groups. By evaluating the results not only for the total collective but also for each stakeholder group, important aspects can be detected that might not be represented in larger expert collectives. By further adapting the questionnaires to the individual stakeholders’ vocabulary or viewpoint, understanding and compliance might be increased and might result in more focused and useful answers. The next steps will be the continuation of the Delphi rounds towards an inter-stakeholder consensus, but more importantly an intra-stakeholder consensus and a step-by-step guide in “good Delphi-method practice”. Further investigations might also help with understanding consensus building in general, for example if some stakeholder groups change their opinions in reaction to other groups.

Some interesting facts were concluded regarding a future IT system for data transfer of documents at end-of-life. Especially security seems to be of high importance, although it was seen as less important by patients than other groups. Maintenance of data by the patients themselves seems to be most preferred, yet medical professionals might not always accept such information, as only 56% of doctors were in favor of a patient-moderated document.

## Supplementary information

**Additional file 1 **Questionnaire Round 0 for *medical professionals*.

**Additional file 2 **Questionnaire Round 0 for *patients*.

**Additional file 3 **Questionnaire Round 1 (exemplaric for *patients*).

## Data Availability

The datasets used and analyzed during the current study are available from the corresponding author on reasonable request.

## Abbreviations

DT: Delphi technique
360°D: 360-Degree Delphi Technique
RWTH Aachen University: Rheinisch Westfälische Technische Hochschule Aachen
SD, stdev: Standard deviation
