# Supplementary material for: 360-degree Delphi: addressing sociotechnical challenges of healthcare IT
Source: BMC Med Inform Decis Mak. 2020 Jun 5;20:101. doi: 10.1186/s12911-020-1071-x (PMC7275570; doi:10.1186/s12911-020-1071-x)
Supplement: Supplementary file 2 — Additional file 2 Questionnaire Round 0 for patients. [file 12911_2020_1071_MOESM2_ESM.docx]

# Additional file 2. Questionnaire Round 0 for *patients*

The following is translated from German. Details of the questionnaire are omitted for readability.

>>Situation description<<

Question 1: How can you communicate your will?

Question 2: How is the described situation a problem for you regarding your will?

Question 3: What problems could occur, if you are not responsive?

Question 4: What misunderstandings can occur regarding your will?

Question 5: How could this situation be improved?

Question 6: What is the basis for the choice of medical therapy?

Question 7: Do you have any other aspects to mention?

>> project description <<

Question 8: What is your opinion of the project?

Question 9: What documents / information would help you?

Question 10: How and where would our project improve the previous situation for you?

Question 11: How and where would our project worsen the previous situation for you?

Question 12: Would you create a data set for yourself or a relative?

Question 13: Which group describes you most precisely?

>> selection of stakeholder groups <<
