# Supplementary material for: 360-degree Delphi: addressing sociotechnical challenges of healthcare IT
Source: BMC Med Inform Decis Mak. 2020 Jun 5;20:101. doi: 10.1186/s12911-020-1071-x (PMC7275570; doi:10.1186/s12911-020-1071-x)
Supplement: Supplementary file 3 — Additional file 3 Questionnaire Round 1 (exemplaric for patients). [file 12911_2020_1071_MOESM3_ESM.docx]

# Additional file 3. Questionnaire Round 1 (exemplaric for *patients*)

The following is translated from German. Details of the questionnaire are omitted for readability. In total 20 questions (sub-questions excluded) were asked, 15 “Simple questions” and 5 matrix questions.

>>project description<<

E-Mail for newsletter? (voluntary)

Question 1: Which stakeholder group do you belong to? (selection of stakeholder groups)

Question 2: Please indicate which documents you know?

Question 3: Please indicate which documents you filled out?

Question 4: What misunderstandings can occur regarding your will?

Question 5: Which of these documents should be online accessible for authorized persons?

Question 6: I do know what happens in an emergency if I’m not able to communicate anymore. (1-4)

Question 7: I have precise wishes should one of these situations happen (y/n)

Matrix questions:

Question 8: Privacy is important to me (1) ➔ Fast data access is important to me (6)

Question 9: Data security is important to me (1) ➔ Fast data inspection is important to me (6)

Question 10: I wouldn’t release all medical data (1) ➔ medical professionals should see data as fast as possible (6)

Question 11: I want lost data to be recovered fast (1) ➔ I want to make sure non-authorized people have no data access (6)

Question 12: I want users to easily edit and see the data (1) ➔ I don’t want non-authorized people to edit or see the data (6)

Question 13: To create an advance directive I want to: Fill out a form, write a free text.

Question 14: I wish there was an easier way to communicate my will in an emergency situation

Question 15: I would create an emergency data set for me. (y/n)

Question 16: Who should be allowed to see my data? (recommendation answers)

Question 17: Who should be able to edit my data? (recommendation answers)

Question 18: How often should the data be updated? (recommendation answers)

Question 19: How much time would you invest in such a data set? (recommendation answers/other)

Question 20: Anything to mention?
